# Supplementary material for: Content-rich biological network constructed by mining PubMed abstracts
Source: BMC Bioinformatics. 2004 Oct 8;5:147. doi: 10.1186/1471-2105-5-147 (PMC528731; doi:10.1186/1471-2105-5-147)
Supplement: Additional File 2 — The original results of the above study (non-essential files are deleted to keep the file size under the limit set by BMC bioinformatics). [file 1471-2105-5-147-S2.bz2 › chilibotAdditionalFile2/dip05/328ID1540130E3910/html/EBGA_EBGC.html]

 


 **EBGA** and **EBGC** 
  
Found 1 abstracts in PubMed, retrieved 1.  
 

 What does Google say? 
 PDF only 
| .edu only 

---

**Interactive relationship** (e.g. stimulation, inhibition, etc)

**Non-interactive relationship** (e.g. studied together, co-existance, homology, etc.)

- The ratio of  **ebgA**  gene product of  **ebgC**  gene product in the functional aggregate of ebg beta galactosidases was determined to be 1 1 by isolation of the enzyme from bacteria grown on uniformly radiolabelled amino acids and separation of the subunits by gel permeation chromatography under denaturing conditions.  Ref: 1540130 Biochem J, 1992
